# Annotations

**Published:** 1897-06-05

**Authors:** 


### Annotations.

### The Poor Man's Hotel.

A decision given last week at the Lambeth Police Court in a summons against the Rowton Houses (Limited) is a matter of serious concern from a public health point of view. It was admittedly a test case brought to decide whether the provisions of the London Building Act, 1894, according to which workmen's dwellings, in narrow streets are not allowed to exceed the width of the street in height, applied to such buildings as the Rowton Houses. Mr. Hopkins dismissed the summons, holding that for the purposes of the Building Act, "he could see no possible difference between, say, the Gordon Hotels Company building a rich man's hotel and Rowton Houses (Limited) building a poor man's hotel." On the face of it this seems a very taking argument. But if it is good law the wording of the Act has certainly failed in its purpose. The magistrate asked on what ground was a restriction to be placed on the one that did not apply to the other. But everyone knows that in many Acts the working classes are protected in regard to their dwellings in a way that no other classes are, and a moment's consideration will show the rottenness of the analogy between such houses as are here in question and high-class hotels. Undoubtedly Rowton Houses are beautiful buildings, as common lodging houses, but it is idle to pretend that in regard to cubic space and other details they can be compared with the Gordon Hotels, and what we have to consider is, not the individual case, but what may be the effect of this decision if acted on by other landowners. The object of the clause in the Act was to limit the height of workmen's dwellings in narrow streets to a height equal to the width of the street. According to this decision, houses such as were here in question can apparently be carried to the full height allowed for other buildings, viz., eighty feet, however narrow the streets in which they stand. We are, then, landed in this dilemma, that if this decision is to be looked upon as generally applicable its effects will be most injurious to the health of the inhabitants of such houses; while, if it is to be looked upon as only applying to the individual case, and if surrounding landlords are not to be allowed to go equally high, its effect is to make a present of a valuable asset to the lucky landlord of this particular plot of land. In any case, the decision is in diametrical opposition to the very object of those who framed the clause, which was to prevent landowners and builders from lining our narrow streets with immense blocks of lofty houses for the working classes; for, so far as health is concerned, it is a matter of indifference whether such houses are lodging-houses or houses let in lodgings.

### Divining Rods Again!

While England generally is crowing so mightily over the advances which have taken place during the past sixty years, it is not uninteresting to observe that, even within about an hour's run of this great metropolis, people are to be found who still believe in the divining rod! Not little ignorant labourers, but people elected by their fellows to represent them on district boards. We read this week of an urban district council which was about to enter upon the work of boring for water, and was applying for a loan of money for the purpose. When, however, it was a question where to bore, instead of consulting an engineer, or a geologist or a surveyor, this notable council must needs consult a "water diviner"! Was there ever such nonsense? Fortunately, the auditor took a common-sense view of the matter, and surcharged the councillors with the payments which they had authorised to the extent of £13 8s. 7d., adding that the courts had held that the pretence of a power, whether moral, physical, or supernatural, was illegal, and that he was bound in the interest of the ratepayers to see that moneys taken from them was not expended on such enterprises as water divining. Among scientific men, undoubtedly, the advances of science have been great; but equally certain is it that outside the pale we are surrounded by a vast sea of ignorance and superstition, and that people are as eager as ever to swallow anything in the shape of quackery, to believe anything that pretends to the supernatural, and to accept the most outrageous statements of the most unblushing charlatans so long as they do but lay claim to powers occult.

### Death in an Hotel.

To die in an hotel is a "very unpleasant occurrence," as is very truly stated in a guide recently issued by an association of Swiss hotel proprietors, and it becomes a matter of no small interest to executors and residuary legatees to know how much it may be legitimately expected to cost. With the object of deciding this knotty point the editor of the *British Medical Journal* has made inquiry at 64 of the best-known hotels at home and abroad concerning the usual customs in case of this "disagreeable occurrence" taking place. The answers received indicate a wide divergence of opinion as to the amount of recompense which is due to the landlord under the circumstances. One good honest man considers that the occurrence of a death is a business risk, and he makes no special charge. Doubtless he knows, as everyone does who has had to do with sickness in an hotel, that the unfortunate patient pays so highly for every little trifle of extra attendance while he lives, that as death does not usually take place suddenly, and severe illness generally brings a number of friends and relatives to the house, the establishment has got its pickings before the end comes, and can well afford to make no special charge for the death itself. Other landlords, however, look on a death as a great opportunity, and a case is mentioned of a visitor to an hotel dying of a wholly non-infectious disorder, in which the landlord's claim for damages (exclusive of the ordinary bill) was upwards of £80. A suggestion is made with apparent approval that a maximum charge should be made of £50 in infectious cases, and £20 in those which are not infectious, where the rooms are charged at 10s. and upwards per day, and half these amounts where the rooms are less than 10s. Our own opinion is that the utmost that the landlord can in equity claim from the representatives of the deceased is for moneys actually expended in carrying out such disinfection and repair as may be ordered or recommended by the medical man or the sanitary authority, together with a reasonable sum for rent and service while these processes are being carried on. We do not think the landlord, after he has finally settled all his little commissions with the tradesmen who do the work, will often find himself out of pocket!

